# Volume 2: Retrospective Analysis of Clinicopathological Characteristics of Surgically Treated Squamous Cell Carcinoma of the Face—A Single-Centre Maxillofacial Surgery Experience

**DOI:** 10.3390/jcm15187218

**Published:** 2026-09-17

**Authors:** Abdullah Saeidi, Shadia Elsayed, Jörg Wiltfang, Christian Flörke

**Affiliations:** 1 Department of Oral and Maxillofacial Diagnostic Sciences, College of Dentistry, Taibah University, Almadinah Almunawarah 42353, Saudi Arabia; ssayed@taibahu.edu.sa; 2 Department of Oral and Maxillofacial Surgery, University Medical Center Schleswig-Holstein, Campus Kiel, 24105 Kiel, Germany; joerg.wiltfang@uksh.de (J.W.); ch.floerke@gmail.com (C.F.); 3 Health and Life Research Center, Taibah University, Madinah 42353, Saudi Arabia

**Keywords:** skin cancer, squamous cell carcinoma, recurrence, histopathology, metastasis

## Abstract

**Background:** Squamous cell carcinoma (SCC) is a common malignancy in Western countries and can lead to extensive tissue destruction, metastasis, and death. The study aimed to highlight the epidemiology, diagnostic patterns, histological variations, and prognostic significance of SCC of the facial skin region based on a single-center analysis. **Methods:** This retrospective study analyzed records of patients admitted to the Department of Maxillofacial Surgery, UKSH-Kiel, for treatment of SCC of the head and face from January 2019 to February 2021. Data were collected via the ORBIS/management-diagnostic codes system (ORBIS-AG, Saarbrücken, Germany). It included information on TNM classification, tumor depth, growth type, tumor localization, and surgical techniques, which were extracted from histopathology and surgical reports. **Results:** A total of 560 patients were admitted for treatment of malignancies of the facial skin region. Sixty patients (10.7%) had SCCs, and their mean age was 80.64 years. A total of 68.3% of the patients were male. The most common histopathological growth type was verrucous: 36.7% (n = 22). No significant correlation was found between growth type and recurrence (*p* = 0.266) or metastasis (*p* = 0.091). However, the correlation between growth type and CT morphology was significant (*p* = 0.032), with radiologically suspicious lymph nodes most frequently associated with a verrucous growth type (36.4%, n = 8/22). The most common locations for SCC were the temporal region (25%, n = 15) and the cheek (15%, n = 9). The majority of SCC cases (32%) were classified as T1, with T4 being the least common (5%). The mean tumor depth for SCC was 4.67 mm (SD: 3.05). Tumor depth showed a significant correlation with TNM classification (*p* = 0.0001). **Conclusions:** This study emphasizes the significance of early detection, accurate advanced T-classifications, tumor depth, and high-risk growth types in predicting recurrence and metastasis in SCC. In this cohort, CT findings were associated with lymph node status and may provide useful information for the preoperative assessment of selected patients.

## 1. Introduction

Squamous cell carcinoma (SCC) accounts for a large fraction of skin cancer cases worldwide, such as in Germany, where 22% of the 213,000 yearly non-melanoma skin cancer (NMSC) diagnoses are SCC [1]. According to an examination of 213,935 cases, the incidence of skin squamous cell carcinoma in Germany increased by more than 500% between 1986 and 2015, with a higher increase in women [2,3]. SCC is the second most prevalent NMSC in North America. In the United States, 2012 data indicated 186,000 to 419,000 new cases in the white population, with men having a higher lifetime risk (9–14%) than women (4–9%) [4,5]. Clinically, Actinic keratosis (Aks) is a recognized precursor lesion for cSCC, with progression occurring in a subset of lesions. On the other hand, beta papillomavirus infection could accelerate the process [6]. This is particularly the case in sun-exposed locations like the head and neck. SCC first manifests as a lump or plaque that can progress into ulcers or necrotic lesions. Excisional biopsy is necessary for a differential diagnosis since SCC might mimic fibroxanthoma, melanoma, or basal cell cancer [7].

The treatment seeks to remove the lesion while preserving skin function and appearance. Surgical excision is the gold standard, with a 95% cure rate. High-risk tumors (>2 cm, >6 mm depth, or with perineural invasion) necessitate broader margins (10 mm) [8]. MOHS micrographic surgery offers thorough margin assessment in anatomically sensitive locations while preserving uninvolved tissue [9]. Radiotherapy may be explored for individuals who are unable or unwilling to undergo surgery in certain situations, depending on tumor features and patient variables. Immunotherapy has been widely used in the treatment of advanced cSCC; nevertheless, treatment response may be impacted by immune resistance mechanisms within the tumor microenvironment [10]. Topical treatments, such as 5-fluorouracil and imiquimod, lack sufficient evidence for SCC treatment due to side effects, dosage difficulties, and low efficacy [11,12].

The prognosis for properly managed cSCC is generally favorable, with reported 5-year cure rates reaching 90% in many cases. The majority of recurrences (75%) occur within the first two years, and metastasis rates remain low (3–5%). Perineural invasion, poor differentiation, and partial excision all increase the likelihood of metastasis, emphasizing the importance of cautious early intervention, the need for individualized treatment approaches, and follow-up [13]. Furthermore, squamous cell carcinoma (SCC) of the skin, especially in the head and neck region, has a high curative rate and low metastatic rates when treated early with complete surgical excision and appropriate adjuvant therapy [14]. Patients may suffer from psychological discomfort since facial skin cancer surgery may leave noticeable scars or alter the appearance of their faces. It is critical to acknowledge these issues while thinking about treatment and postoperative care. Early identification and comprehensive support are essential for improving the quality of life of these patients [15,16].

Therefore, in a single-center cohort, this study aimed to describe the clinicopathological features of surgically treated facial cSCC and investigate the relationships between histopathological development patterns, tumor characteristics, CT findings, recurrence, and lymph node metastasis based on analysis from a single center in Germany.

## 2. Materials and Methods

### 2.1. Study Design

This retrospective single-center study was carried out at the Department of Oral and Maxillofacial Surgery, University Medical Center at Schleswig-Holstein (UKSH), Kiel, between January 2019 and February 2021. The study design, clinical setting, inclusion and exclusion criteria, and surgical technique were all identical to those detailed in our earlier publication on basal cell carcinoma [17]. Data records were included after histological confirmation of squamous cell carcinoma, and each case was treated using the same standardized surgical approach. Medical records for patient follow-up are accessible for a minimum of three years. As part of follow-up exams, the timing of secondary tumors and tumor recurrences (local, lymph node, and distant metastases) were noted. After determining disease-free survival (DFS), all data was anonymized and moved to an Excel spreadsheet.

### 2.2. Inclusion and Exclusion Criteria

Inclusion requirements included: (1) Histopathological confirmation of at least one primary facial SCC; (2) a tumor that was treated surgically only. Exclusion criteria were: (1) Incomplete data in patients’ records; (2) patients treated with non-surgical therapy; (3) incomplete therapy/or death; and (4) associated head malignancy.

### 2.3. Data Collection Technique

**Figure 1:**
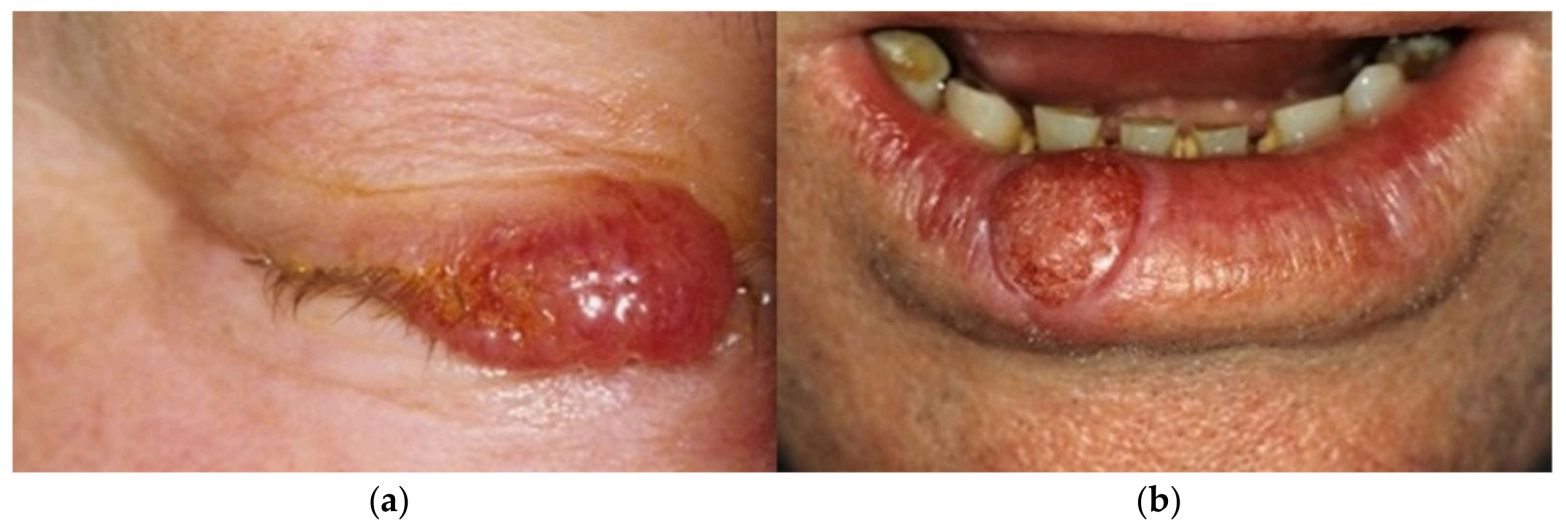
Squamous cell carcinoma in the upper lid (**a**) and lower lip (**b**).

A separate Excel sheet was used to collect information on TNM status, tumor size, tumor depth, type, tumor localization, metastasis, recurrence, and surgical procedures, as well as patients’ demographic data, such as age and gender (Figure 1). In accordance with the Union for International Cancer Control’s (UICC) categorization guidelines, the histopathology report provided the TNM status information.

### 2.4. Cutaneous Squamous Cell Carcinoma Histopathological Evaluation

Histological growth types were assessed for each tumor, ranging from well-differentiated to poorly differentiated variants. Variants included low-risk types (e.g., keratoacanthoma, verrucous SCC) and high-risk types (e.g., spindle cell SCC, desmoplastic SCC). Uncommon variants such as sarcomatoid, lymphoepithelioma, giant cell osteoclast-like SCC, and pseudovascular SCC were also recorded.

### 2.5. Lymph Node Metastasis

Metastatic involvement in lymph nodes was evaluated using the anatomical levels I–V classification, based on the Shah et al. 1981 system [18]. We identified a particular abnormal CT morphology of lymph nodes that appeared suggestive of metastatic involvement due to their unusual size and form. Radiologically suspicious cervical lymph nodes were referred to as “abnormal CT morphology,” which did not suggest bone invasion. When lymph nodes showed characteristics like increased size, rounded shape, heterogeneous enhancement, central necrosis, loss of the usual nodal architecture, or irregular margins/extranodal extension, they were deemed radiologically suspicious in our retrospective evaluation. Although these radiological results were utilized to detect possible cervical lymph node metastases, the final standard for confirming lymph node metastasis was thought to be histopathological analysis. Tumor behavior was examined in relation to lymphatic drainage patterns and metastatic pathways.

### 2.6. Statistical Analysis

Data analysis was done using IBM SPSS Statistics version 27.0. All variables were subjected to descriptive statistics; for continuous data, means and standard deviations were reported, while for categorical data, frequencies and percentages were reported. Fisher’s exact test was used to investigate associations between categorical variables, and the independent *t*-test was used to evaluate differences between two independent groups. Statistical significance was defined as a *p*-value of less than 0.05.

## 3. Results

### 3.1. Patient Demographics

A total of 560 individuals were admitted for treatment of skin cancers affecting the head and face. After applying the inclusion and exclusion criteria, 185 patients met the criteria for inclusion in this retrospective analysis of non-melanoma skin cancer. Of these, 125 were identified with basal cell carcinoma, and 60 with cutaneous squamous cell carcinoma. The average age of patients with squamous cell carcinoma was 80.64 years; a total of 68.3% were male.

### 3.2. Tumor Classification and Distribution of cSCC

Carcinoma in situ: 8% (n = 5), T1: 32% (n = 19), T2: 30% (n = 18), T3: 25% (n = 15), and T4: 5% (n = 3). The majority of cSCC cases (32%) were classified as T1, with T4 being the least common (5%) (Table 1).

Regarding the relation between T-Classification and recurrence in cSCC, we have the following: (1) Four (26.7%) out of 15 patients diagnosed with cSCC and classified as T3 had tumor recurrence; (2) one (33.3%) out of three patients diagnosed with cSCC and classified as T4 had tumor recurrence; and (3) no patient diagnosed with cSCC who was classified as carcinoma in situ had tumor recurrence (Table 1) (*p*-value = 0.002).

Regarding the relation between T-Classification and cSCC metastasis, we have the following: (1) One (5.3%) out of 19 patients classified as T1 had tumor metastasis; (2) two (11.1%) out of 18 patients classified as T2 had tumor metastasis; (3) four (26.7%) out of 15 patients classified as T3 had tumor metastasis; and (4) one (33.3%) out of three patients classified as T4 had tumor metastasis. No patient classified as having carcinoma in situ had tumor metastasis (Table 1). A statistically significant association was observed between TNM classification and metastases among tumors that were diagnosed with SCC (*p*-value = 0.027).

**Chart 1:**
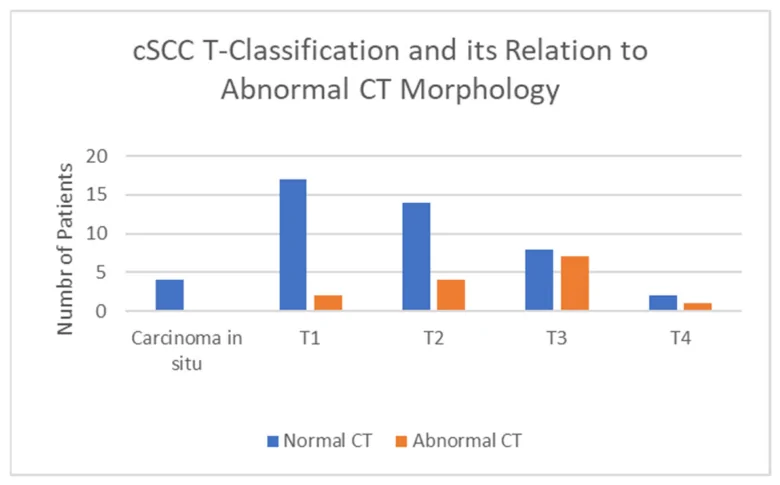
SCC T-classification and its relation to abnormal CT morphology.

Regarding the relationship between T-classification and abnormal CT morphology among cSCC patients, approximately 75% of patients (n = 45) had normal CT morphology. The suspected CT morphology among T-classification was distributed as follows: seven out of fifteen patients classified as T3 had abnormal CT morphology (four of them at levels 1 and 2 on one side). Four patients classified as T2 had abnormal CT morphology (two of them at levels 1, 2, and 3 on one side, with the other two at levels 1, 2, and 3 on both sides). One out of three patients, classified as T4, had an abnormal CT morphology at levels 1, 2, and 3 on one side. There was a highly significant correlation between T-classification and abnormal CT morphology in patients diagnosed with cSCC (*p*-value = 0.001) (Chart 1).

### 3.3. Tumor Location on the Face for Patients Diagnosed with cSCC

Table 2 shows the anatomical distribution of the lesions. The most common cSCC locations on the face were the temporal region (n = 15, 25%) and the cheek (n = 9, 15%). There was no significant correlation between tumor location and recurrence or metastases among SCC-diagnosed patients (*p* ≥ 0.05).

### 3.4. Histopathology and Cancer Growth Type (cSCC)

Verrucous was the most common histological category among the 60 patients with SCC diagnoses. Overall, 14 individuals exhibited CT-suspected metastatic lymphadenopathy, with significant differences between subtypes (*p*-value = 0.032). Two patients had acantholytic growth type; both of them had CT morphology with suspected lymph node metastasis. Twenty-two patients had verrucous growth type; eight of them (36.4%) had CT morphology with radiologically suspicious lymph nodes. Fifteen patients had desmoplastic growth type; three of them (20%) had CT morphology with radiologically suspicious lymph nodes. Twelve patients with spindle growth type (8.3%), including one of them had CT morphology with suspected lymph node metastasis. No suspected lymph node metastasis was noted in the keratoacanthomas and superficial growth type (Table 3).

### 3.5. Growth Type and Its Relation to the Presence of Positive Lymph Nodes

Positive lymph nodes were found only in the desmoplastic, spindle, and verrucous growth types. Five (22.7%) out of 22 patients had the verrucous growth type and one or more positive lymph nodes after neck dissection. In the spindle and desmoplastic growth types, only one patient in each type (8.3% and 6.7%, respectively) had one or more positive lymph nodes after neck dissection. The correlation between positive lymph nodes and growth type indicated that there was no statistically significant correlation, although it did not reach the prespecified significance threshold (*p* = 0.056). No statistically significant correlation was found between growth type and recurrence or metastasis among SCC (*p*-value ≥ 0.05).

### 3.6. Tumor Depth and the Presence of a Positive Lymph Node

The mean infiltration depth was 4.67 mm with a standard deviation of 3.05. A total of 18.3% of patients with a mean tumor depth of 3.0 mm (n = 11) were classified as T1 (n = 8). The highest mean tumor depth of 10 mm was observed in the T4 classification (n = 2), while the lowest mean tumor depth of 1.0 mm was observed in only one patient in the T1 classification. There was a significant correlation between tumor depth and TNM classification (*p*-value = 0.0001).

Positive lymph node variables were categorized as 0 = no positive lymph nodes and 1 = presence of positive lymph nodes. A total of 88.3% of cSCC patients had no positive lymph nodes (n = 53). The mean infiltration depth among tumors with positive lymph nodes was 6.4 mm. A total of 18% of the patients had a mean infiltration depth of 3 mm with no positive lymph nodes (n = 11). No correlation was found between tumor depth and positive lymph nodes among cSCC tumors.

### 3.7. Tumor Depth and Recurrence Among cSCC Patients

Five patients had recurrence (8.3%), while the rest of the patients (91.7%) had no recurrence. Eighteen percent of patients had a tumor depth of >9 mm (n = 11), the same percentage of patients with an infiltration depth of 3 mm. The correlation between tumor depth and recurrence was statistically insignificant among cSCC patients.

### 3.8. Tumor Depth and Metastasis Among cSCC Patients

The metastasis variable was categorized as 0 = no metastasis and 1 = metastasis present. A total of 86.7% of cSCC patients had no metastasis (n = 52). The mean infiltration depth among tumors with metastasis was 5.87 mm. Eighteen percent of patients had 3 mm average infiltration depth and no metastasis. No correlation between infiltration depth and metastasis was found at a significance level of 0.05.

### 3.9. CT Morphology and Its Relation to Positive Lymph Nodes

A total of 75% of patients had no CT findings or were normal. Seven patients (11.7%) had suspected CT morphology and positive lymph nodes. Eight patients with suspected CT morphology had negative lymph nodes. There was a highly statistically significant correlation between the abnormal CT morphology and the presence of positive lymph nodes among cSCC-diagnosed patients (*p*-value = 0.001).

## 4. Discussion

This study investigated the pathological, demographic, and clinical aspects of cutaneous squamous cell carcinoma (cSCC) treated at the Department of Oral and Maxillofacial Surgery, UKSH, Kiel. It focused on incidence, recurrence, metastasis, T-classification, growth types, and their relation to tumor characteristics and imaging. The findings align with global trends and contribute to the knowledge base. In the current study, 60 patients had squamous cell carcinoma. Around 68.3% of the patients were male. The mean age at the time of admission was 80.64 years, which is in accordance with previous reports of the Institute of Cancer Epidemiology at the University of Luebeck, Germany [17]. They have noticed that males are more affected than females, and the mean age of all non-melanoma skin cancer patients was 70 years [19]. In their investigation of 232 patients with non-melanoma skin cancer, Rahimi-Nedjat et al. 2021 [20] found similar results regarding age and gender, with a slight difference. A total of 69.8% of the patients were male. The average age was 77.1 years (±11.3 years) [20].

In the present study, the recurrence among patients diagnosed with cSCC was five patients (8.3%) out of 60. Chren et al. 2011, in their prospective cohort study, studied 608 primary non-melanoma skin cancer cases between the years 1999 and 2000 and found that 21.5% of 608 non-melanoma skin cancer cases recurred (including BCC and cSCC) [21]. Domnguez-Cherit et al. (2017) followed 103 patients with cSCC for ten years and found that only four patients (3.8%) had cSCC recurrence in the first and fourth years after the first therapy [22]. The cSCC recurrence rate in this study is relatively high in comparison to the published articles.

Eight (13.3%) out of 60 patients diagnosed with cSCC had tumor metastasis. Venables et al. (2019) reviewed the files of 76,977 patients in England diagnosed with primary cSCC between 2013 and 2015; they discovered that most metastases (85%) occurred within the first two years of the primary diagnosis; a total of 1566 patients had cSCC metastasis out of 76,977 [23]. Comparing data from previous articles to the current results suggests that differences in the metastasis rates are due to how specialized the treatment center is and whether they treat all skin changes or only treat severely complicated and late stages of cSCC.

A large-scale study from Birmingham reviewed a total of 680 cSCC patients. A total of 523 tumors were classified as T1 (76.9%), 36 cases were classified as T2 (5.3%), 119 cases were classified as T3 (17.5%), and only 2 cases had T4 (0.3%) [24]. In the present study, the majority of the patients in Kiel were classified as T1 (32%). Eighteen patients (30%) were classified as T2, fifteen patients (25%) were classified as T3, and three patients (5%) were classified as T4. The data in this study and in the articles mentioned support the theory that T1 is the most common classification for cSCC, with a significant correlation between tumor diagnosis and T-classification (*p* = 0.002).

A study conducted at Toulouse University in 2014 found recurrence in 15.21% of 69 patients diagnosed with cSCC. All of them were classified as T3 [25]. The Birmingham study reviewed a total of 459 patients with 680 cSCC and found that all tumors classified as T4 (0.3%; n = 2) had recurrence, and most of the recurrences were in tumors classified as T3: 20 cases out of 119. Recurrence also existed among patients classified as T1 and T2: seven cases and five cases, respectively [24]. In this study, four patients out of 15 classified as T3 (26.7%) had tumor recurrence; one patient (33.3%) out of three classified as T4 had tumor recurrence; and there was no recurrence among the other classifications. There was a highly significant correlation between T classification and recurrence among tumors that were diagnosed as SCC (*p*-value = 0.002). The data oppose the theory, indicating that recurrence is more common in large tumors and can occur in T2 or even T1 cases.

A study from Japan reviewed 540 cSCC cases. A total of 4.4% of the cases had at least one lymph node metastasis. One hundred and six patients were classified as T3; a total of 23 cases (21.7%) had a lymph node metastasis, and one patient classified as T4 had lymph node metastasis [26]. In this study from Kiel, one patient (5.3%) out of 19 classified as T1 had tumor metastasis. Two patients (11.1%) out of 18 classified as T2 had tumor metastasis. Four patients (26.7%) out of 15 classified as T3 had tumor metastasis. One patient (33.3%) out of three classified as T4 had tumor metastasis. The correlation was highly significant between T classification and metastases among tumors that were diagnosed as SCC at the alpha level of 0.05 (*p*-value = 0.027). The data from previous studies support the theory that tumor size is highly correlated with lymph node metastasis.

In this study, the most frequent location for cSCC on the face was the temporal region (n = 15; 25%), followed by the cheek region with nine cases (15%), the scalp with seven patients (11.7%), and the lower eyelid with six patients (10%). The least common region was the upper lip, with only one patient (1.7%). Ciuciulete et al. 2021 also reviewed 103 cases of cSCC; a total of 76.7% of the cases were in the head and neck region—unfortunately, both articles did not study the head and neck areas in detail [27]. Feinstein et al. (2019) studied 53 cSCC cases between 2006 and 2017, and 49.1% of the cases occurred in the face; nine cases out of the 53 occurred in the scalp at 17%, followed by eight cases in the temporal region at 15%, seven cases in the cheek region at 13%, four cases at 7.5% in the forehead and in the nose, respectively [28]. Remenschneider et al. (2015) studied cases of 47 patients diagnosed with cSCC in the head and neck region and found that the most common areas for cSCC were the cheek region, at 31.9% (n = 15), and the scalp region, at 27.7% (n = 14) [29]. They also found that the nose and the lip regions were the least common areas, with only one patient for each region. Data from previous articles suggested that the head and neck regions were the most common sites in the body for cSCC. The data also supported the theory that the scalp, temporal, and cheek regions, including the periauricular area, were the most common sites for cSCC on the face.

Ci et al. (2022) reviewed 103 cases of cSCC in Romania; a total of 75.8% of the cases were described as conventional histopathological type; a total of 9.7% of the cases were described as acantholytic growth type, followed by the verrucous growth type at 7.8%; two cases were described as spindle (1.9%), and one case was described as desmoplastic (1%) [30]. Chabrillac et al.’s 2019 study reviewed the relation between growth type and lymph node positivity; they found no association between the histological growth type and lymph node positivity [25]. A clear theory combining these two factors cannot be established due to the scientific studies’ deficiency, so further investigation is recommended. Meanwhile, studies have found that growth types with aggressive behavior, such as verrucous, desmoplastic, spindle, and acantholytic, have double the risk of metastases. Their metastasis potential was 21.4–44% [31,32]. Desmoplastic and spindle cSCC growth types have shown six times more potential for metastasis [33]. Previous studies opposed the theory that no correlation has been found between growth types and metastases. More cases should be investigated. The cases in this study were not enough to oppose their theory.

Lee et al. 2016 collected 26 cSCC cases for their prospective study and measured the depth and its relation to other factors. They found that the mean invasion depth was 7.2 mm and the range was 2–25 mm [34]. Tang et al. 2018 [35] studied 45 cSCC cases, and the mean depth in the head and neck regions was 2.28 mm, and the range was 0.5–6 mm. They concluded that the biopsy should be at least 3 mm to capture the tumor invasion [35]. The tumor depth in cSCC can range from 1 mm to more than 20 mm. The mean can range between 2 and 7 mm with regard to T-classification and tumor depth. Lee et al. 2016 indicated that tumor depth is significantly correlated with tumor size, and that tumor depth will increase accordingly with the increase in T-classification [34].

Oh et al. 2020 found a strong correlation between cSCC invasion and recurrence [36]. Multiple studies have found that the vertical tumor depth is a high-risk factor for cSCC recurrence [37,38]. All the previous articles oppose our result of no correlation between the depth and cSCC recurrence due to the small amount of the sample size in this study and the short follow-up period. Roozeboom et al. 2013 mentioned that a 1 mm increase in tumor thickness increases the risk of lymph node metastases by 5% to 20% [39]. Schmults et al. 2013 mentioned that cSCC depth beyond the subcutaneous fat is highly correlated with lymph node metastasis [40]. These findings differ from previous reports of there being no correlation between depth and cSCC metastasis because of the small sample size in this study and the short follow-up period. Therefore, more patients should be included in further studies.

Remenschneider et al. 2015 performed 92 CPECT/CTs followed by sentinel lymph node biopsy on 47 patients with skin cancer in the head and neck regions, and they found that the ability of CT to identify positive lymph nodes or the adjacent structure was 92% [29]. Sethi et al. 2018 [41] studied 53 patients with head and neck cutaneous malignancy who underwent SPECT/CT and lymph node biopsy. The CT sensitivity was 69.2%, and the specificity was 88.9%, with a positive predictive value of 85.7% [41]. Previous research supports the use of preoperative CT to guide the surgeon during decision-making and intraoperatively.

The limitations of the present investigation include a retrospective design, which limits the ability to account for missing data and incomplete follow-up of patients who were unable to be followed up with or who had died. Further studies with larger cohorts and extended follow-up periods are necessary to provide a comprehensive understanding of NMSC prognosis. However, this study emphasizes the significance of early detection, accurate advanced T-classifications, tumor depth, and high-risk growth types in predicting recurrence and metastasis in cSCC. Preoperative imaging, particularly CT, is an effective tool for guiding surgical decisions and assessing lymph nodes involvement. 

## 5. Conclusions

The present findings align with global trends and contribute to the knowledge base. No significant correlation was found between growth type and recurrence or metastasis. There was a significant correlation between SCC tumor type, CT morphology, and lymph node positivity. In this cohort, CT findings were associated with lymph node status and may provide useful information for the preoperative assessment of selected patients.

## Figures and Tables

**Table 1:** T-Classification distribution among cSCC patients and recurrence in patients diagnosed with cSCC.

Outcome	Number of Patients (n)	Percentage (%)	T-Classification	Number of Patients (n)	Percentage (%)	Recurrence (n)	Recurrence Rate (%)	Metastasis (n)	Metastasis Rate (%)
Tumor recurrence	5	8.3	Carcinoma in situ	5	8	0	0	0	0
Tumor metastasis	8	13.3	T1	19	32	0	0	1	5.3
No recurrence	55	91.7	T2	18	30	0	0	2	11.1
No metastasis	52	86.7	T3	15	25	4	26.7	4	26.7
			T4	3	5	1	33.3	1	33.3

**Table 2:** Anatomical distribution of cutaneous squamous cell carcinoma (cSCC) lesions (n=60).

Anatomical Region	Number of Patients (n)	Percentage (%)
Temporal	15	25.00%
Cheek	9	15.00%
Scalp	7	11.70%
Lower lid	6	10.00%
Nose	5	8.30%
Forehead	4	6.70%
Eyebrow	3	5.00%
Medial eye corner	3	5.00%
Lower lip	3	5.00%
Nasolabial	2	3.30%
Upper lid	1	1.70%
Lateral eye corner	1	1.70%
Upper lip	1	1.70%
Total	60	100.00%

**Table 3:** Suspected CT morphology and growth type in cSCC.

Histopathological Growth Type	Total Cases, n (%)	CT Suspected Malignant Nodes, n (%)
Verrucous	22 (36.7%)	8 (36.4%)
Desmoplastic	15 (25.0%)	3 (20.0%)
Spindle	12 (20.0%)	1 (8.3%)
Keratoacanthoma	3 (5.0%)	0 (0.0%)
Superficial	2 (3.3%)	0 (0.0%)
Acantholytic	2 (3.3%)	2 (100.0%)
Not specified	4 (6.7%)	—
Total	60 (100.0%)	14 (23.3%)

## Data Availability

The data presented in this study are available on request from the corresponding author.
